# Remodeling Alzheimer-amyloidosis models by seeding

**DOI:** 10.1186/s13024-021-00429-4

**Published:** 2021-02-15

**Authors:** Brittany S. Ulm, David R. Borchelt, Brenda D. Moore

**Affiliations:** Department of Neuroscience, Center for Translational Research in Neurodegenerative Disease, McKnight Brain Institute, College of Medicine, University of Florida, Gainesville, FL USA

**Keywords:** Alzheimer’s disease, Seeding, Amyloid-β, Mouse models, Prion disease

## Abstract

Alzheimer’s disease (AD) is among the most prevalent neurodegenerative diseases, with brain pathology defined by extracellular amyloid beta deposits and intracellular tau aggregates. To aid in research efforts to improve understanding of this disease, transgenic murine models have been developed that replicate aspects of AD pathology. Familial AD is associated with mutations in the amyloid precursor protein and in the presenilins (associated with amyloidosis); transgenic amyloid models feature one or more of these mutant genes. Recent advances in seeding methods provide a means to alter the morphology of resultant amyloid deposits and the age that pathology develops. In this review, we discuss the variety of factors that influence the seeding of amyloid beta pathology, including the source of seed, the time interval after seeding, the nature of the transgenic host, and the preparation of the seeding inoculum.

**Supplementary Information:**

The online version contains supplementary material available at 10.1186/s13024-021-00429-4.

## Background

Alzheimer’s Disease (AD) is pathologically defined by the coexistence of β-amyloid (Aβ) deposits and neurofibrillary tangles [1]. Given the role of Aβ deposition in AD postmortem diagnosis, generating mouse models that reproduce this pathology by expressing mutant amyloid precursor protein (APP) or presenilin 1 (PS1) has been a substantial focus of research efforts (reviewed in [2]). Mice that express multiple transgenes related to familial AD, mutant APP and PS1, can develop pathology relatively early in life (3–6 months). Conversely, there are models that solely express human APP (wild-type or mutant) that develop amyloid pathology later in life (12–18 months) [3]. Seeding has been shown to accelerate the time-course and severity of Aβ deposition in various APP transgenic models, as well as influence the morphology of the ensuing deposits (Tables 1 and S1). These findings in mice parallel early seeding studies that were done in non-human primates, where Aβ deposition was induced (generally after 10ys of age) by seeding from human brain tissue [23–27]. The induced deposits and cerebral angiopathy were detected by immunohistochemistry, Congo red, and silver staining [25, 27]. Seeding has similarly been shown to accelerate pathology caused by the misfolding of tau and α-synuclein in mouse models (reviewed in [28–30]). In this review, we focus on the variables at play in manipulating Aβ deposition in APP transgenic mice by seeding.

**Table 1:** Summary of selected Aβ seeding studies

Reference	Source of seed (route of injection)	Host Line	Host Onset	Host Pathological Features	Age Observed (Age at Time of Injection)	Pathological Features of seed	Pathological Features of Induced Aβ Pathology
[4]	Human (IHC)	Tg2576	~ 9 months	Mixed core > diffuse	8 months (3 months)	“plaques and Neurofibrillary tangles”	Primarily diffuse deposits, largely Aβ42
[5]	Human (IHC)	Tg2576	~ 9 months	Mixed core > diffuse	8 and 15 months (3 months)	“plaques and Neurofibrillary tangles”	Primarily diffuse in cortex with some cored deposits in corpus callosum
[6]	Human (IHC)	APP23	6–9 months	Mixed core > diffuse	9 months (5 months)	“plaques and neurofibrillary tangles”	Significant Aβ deposition that appeared diffuse
	Mouse (APP23) (IHC)				7–9 months (5 months)	Fibrillar, congophilic, Aβ40 > Aβ42	“Diffuse and filamentous”. Some congophilic parenchymal deposits near vessels. Dystrophic neurites.
	Mouse (APPPS1) (IHC)				8 months (5 months)	Fibrillar, congophilic, Aβ42 > Aβ40	“course, punctate”
	Synthetic Aβ and WT brain extract						Limited deposition, similar to below
	Synthetic Aβ42 (100-1000X) (IHC)				9 months (5 months)	Fibrillar, congophilic	Some deposits in dentate gyrus, amorphous mass (largely injectate)
	Mouse (APP23) (IHC)	APPPS1	3–6 months	Mixed core > > diffuse	3 and 5 months (2 months)	Fibrillar, congophilic, Aβ40 > Aβ42	“mixture of filamentous and compact”
	Mouse (APPPS1) (IHC)					Aβ42 > Aβ40	“course and punctate deposition”
[7]	Mouse (APP23) (Varied: Olf Bulb, parietal cortex, entorhinal cortex, striatum, IHC)	APP23	6–9 months	Mixed core > diffuse	3 months post injection (2–5 months, proximal to injection site), more robust 6 months after injection.	Fibrillar, congophilic, Aβ40 > Aβ42	Parenchymal diffuse Aβ with variable congophilic core plaques and vascular deposits (see Table S1 for more details).
[8]	Mouse (APP23) IP injection	APP23	6–9 months	Mixed core > diffuse	8–9 months (2 months)	Fibrillar, congophilic, Aβ40 > Aβ42	CAA carrying into nearby parenchyma. Congophilic vascular Aβ, surrounded by “diffuse, Congo red-negative Aβ deposits”.
[9]	Mouse (APP23) (IHC)	Tg (APP23:Gfap-luc)	6–9 months	Mixed core > diffuse	12 months (~ 2 months)	Fibrillar, congophilic, Aβ40 > Aβ42	“large numbers of small Aβ plaques … [and] more diffuse Aβ deposits”.
[10]	Human (IHC)	human WT APP Hetero zygotes (HuAPPwt)	N/A	N/A	450 days, 615 days, 750 days (165 days)	“plaques and Neurofibrillary tangles”	Diffuse deposits (450 and 615 days). 3/7 mice ThioS positive (750 days).
[11]	Mouse (APP23, fractionated proteinase K treated) (IHC)	APP23	6–9 months	Mixed core > diffuse	7–9 months (3–4 months)	Fibrillar, congophilic, Aβ40 > Aβ42	Mixture diffuse and congophilic deposits. Aβ deposits small and punctate, some congophilic.
	Mouse (APP23, M or F, extra-sonicated) (IHC)						
[12]	Mouse (APP23) (IHC)	R1.40 APP (homozygous)	~ 15 months	Mixed core > diffuse	9 months (3 months)	Fibrillar, congophilic, Aβ40 > Aβ42	Largely diffuse deposits in parenchyma and near vessels.
					15 months (9 months)		
					15 months (3 months)		
[13]	Mouse (APP23, CRND8) (ICV)	APP23:Gfap-luc	6–9 months	Mixed core > diffuse	330–385 days post injection (2 months)	Fibrillar, congophilic	Increased Aβ deposition; morphology not described.
	Brain purified fibrils (ICV)					Fibrils. 15-20x more Aβ-rich than non-purified	Increased Aβ deposition; morphology resembles cored plaques.
	Synthetic WT Aβ40 (ICV) Synthetic S26C Aβ40 (ICV)					Fibrillar, congophilic	Increased Aβ; morphology resembles cored plaques in corpus callosum.
[6, 14, 15]	Mouse (APP23) (IHC)	APP23	6–9 months	Mixed core > diffuse	7–9 months (4–6 months)	Fibrillar, congophilic, Aβ40 > Aβ42	Diffuse deposits in molecular layer of dentate gyrus that exhibit spectral properties of seed source when stained with trimeric polythiophene acetic acid
	Mouse (APPPS1) (IHC)					Fibrillar, congophilic, Aβ42 > Aβ40	
	Mouse (APP23) (IHC)	APPPS1 mice	3–6 months	Mixed core > > diffuse	3–4 months (1.5–2 months) and 6 months (3 months)	Fibrillar, congophilic, Aβ40 > Aβ42	
	Mouse (APPPS1) (IHC)					Fibrillar, congophilic, Aβ42 > Aβ40	
[16]	Synthetic Aβ40 (NaP) (ICV)	APP23:Gfap-luc	6–9 months	Mixed core > diffuse	330 days after injection (6–8 weeks)	(long straight fibrils, rarely short fibrils)	Mixed ThioS-positive compact deposits with dense Thio-S negative deposits.
	Synthetic Aβ42 (NaP) (ICV)					(long fibrils, mostly short fibrils)	
	Synthetic Aβ40 (NaP/SDS)					(long straight fibrils)	
	Synthetic Aβ42 (NaP/SDS)					(long fibrils with some twists)	
[17]	Mouse (APP23/APPPS1) (IP injection)	APP23	6–9 months	Mixed core > diffuse	7–8 months (1–2 months), more robust 7–8 months after injection	Fibrillar, congophilic,	Diffuse parenchymal, variable vascular deposition. 5–15% deposits congophilic.
	Mouse (APP23/APPPS1) (IP injection)	homozygous R1.40	10–14 months	Mixed core > diffuse	Somewhat at 9–10 months (1–2 months), more robust 10–12 months after injection.	Fibrillar, congophilic,	Deposits of neocortex in younger groups largely vascular, but in older groups more parenchymal diffuse plaques.
	Mouse (APP23/APPPS1) (IP injection)	hemizygous APP23 with murine APP −/−	9–10 months		9–10 months (1–2 months)	Fibrillar, congophilic,	Diffuse parenchymal, vascular deposits evidence with 5% congophilic.
[18]	Human (fixed) (IHC)	APP23	6–9 months	Mixed core > diffuse	7–8 months (3–4 months)	“plaques and neurofibrillary tangles”	Small, compact, punctate Aβ deposits. Thioflavin-S and Congo Red staining not reported.
	Mouse (APPPS1, fresh frozen) (IHC)					Fibrillar, congophilic,	
	Mouse (APPPS1, fixed and cryoprotected) (IHC)						
	Mouse (APP23, fresh frozen) (IHC)						
	Mouse (APP23, fixed and cryoprotected) (IHC)						
[19]	Human (fresh frozen supernatant from formic acid-soluble fraction) (IHC)	APP23	6–9 months	Mixed core > diffuse	12 months (4 months)	“plaques and Neurofibrillary tangles”	Diffuse Aβ depositions.
	Human (supernatant mixed with CSF) (IHC)				10–11 months (3–4 months)	“plaques and Neurofibrillary tangles”	Robust Aβ deposition, largely diffuse.
[20]	Mouse (APP23 mouse hippocampi seeded 1 and 30 days prior with APP23 brain tissue)	APP23 mice (male)			7 and 11 months (3 months)		Some congophilic deposits, both parenchymal and vascular.
[21]	Mouse (APP23) (IHC)	APP23 mice	6–9 months	Mixed core > diffuse	Robust at 9–10 months (3–4 months)	Fibrillar, congophilic, Aβ40 > Aβ42	“Diffuse and filamentous” Aβ deposition.
	Mouse (APPPS1) (IHC)				Robust at 9–10 months (3–4 months)	Fibrillar, congophilic, Aβ42 > Aβ40	“Punctate and compact” Aβ deposition
[22]	Mouse (5xFAD) (IHC)	5xFAD	~ 4 months (hippocampus)	Mixed core > > diffuse	13 weeks initial plaques observed; also 4 months (7 weeks)	Fibrillar, congophilic, Aβ42 > Aβ40	Punctate and compact deposits in hippocampus and dentate gyrus
	Mouse (APP23) (IHC)	APP23	6–9 months		9 months (6 months)	Fibrillar, congophilic, Aβ40 > Aβ42	

One of the most notable variables between different Aβ seeding paradigms is the source of the seed, which can be grossly separated into recombinant peptides aggregated in vitro, human donor tissue homogenates, and transgenic mouse model tissue homogenates; each of which have their own nuances that influence the induced pathology. The selection of the host model that has been challenged with Aβ seeds can also factor in the pathological outcomes. Together, these variables conspire to modulate the type of Aβ pathology that is induced by seeding.

The intracerebral injection of tissue homogenates or purified Aβ seeds would be expected to induce gliosis that could also influence amyloid seeding. The induction of gliosis alone does not seem to be sufficient to induce Aβ deposition as injection of human brain lysates from aged individuals with small amounts of Aβ pathology results in little to no seeding [6]. Additionally, previous work by Chakrabarty et al., has shown that when gliosis is chronically activated in the brains of APP transgenic mice by overexpression of the cytokine IL-6, Aβ deposition is attenuated [31]. Further, environmental enrichment of the 5xFAD mice resulted in increased levels of activated microglia and a concomitant reduction of Aβ seeding [22]. Together these results indicate that any inflammation that arises in these models from injection of Aβ seed preparations could diminish seeding efficacy. However, the overwhelming evidence from published studies indicate that if inflammation is induced by the injection of seeds, then the activity of the injected Aβ seeds is sufficient to overwhelm any negative effects of inflammation that may have been induced.

One of the most consistent hallmarks of Aβ seeding is the acceleration of pathology. Bilateral hippocampal (and overlying neocortical) injection of 5-month-old APP23 mice with brain homogenates from aged APP23 mice results in hippocampal seeding of Aβ deposition 1 month earlier than uninjected mice [6]. Accelerated deposition in APP23 mice is also observed when the seeding homogenate is derived from other murine amyloid-depositing models such as APPPS1 mice [6]. Similarly, young APPPS1 mice injected with brain extracts from older APP23, or APPPS1, mice show earlier onset of amyloidosis [6]. This acceleration of pathology may be appreciable only near the site of seed injection, as described in APP23 mice that received intrahippocampal injections at 4–6 months of age to then develop pathology at 7–9 months of age [14]. Notably, in this paradigm other regions of the brain may already be showing pathology by the age of analysis, indicating that injected seeds are introduced into a CNS with elevated levels of total Aβ burden.

The role that aging plays in mediating the severity of seeded Aβ pathology has been difficult to address because nearly all of the studies have used mice that will eventually develop Aβ pathology. In a study in the R1.40 APP transgenic mice that do not develop pathology until 15 months of age, injection of seeds at 3 or 9 months produced the same levels of pathology at 6 months post-injection [12]. Aging the mice for 12 months after seeding resulted in a widespread distribution of amyloid pathology throughout the forebrain, with congophilic plaques proximal to the injection site [12]. Collectively, these findings indicate that severity and onset of Aβ pathology in APP transgenic mice can be effectively accelerated by injecting young animals with brain extracts from older mice with high levels of Aβ pathology.

## Seeds derived from aged APP transgenic mice

One of the benefits of using seeds prepared from existing transgenic mouse models is the ability to select for a vast range of variables, including genotype, age, sex, and fixation methods, in addition to other post-harvest processing methods. Additionally, humans with AD display a spectrum of Aβ pathologies ranging from diffuse, to vascular, to cored neuritic deposits [32]. Although many mouse models show a similar spectrum of pathology, a subset of models that have been described show a preponderance of one type of Aβ pathology over another [33]. For example, the APPswe/PS1dE9 models of amyloidosis are prone to develop cored neuritic Aβ deposits early [33, 34]. Mice that express a mutant murine APP develop Aβ deposits that are primarily diffuse [33]. Thus, it may be possible to manipulate the type of Aβ pathology that is seeded by selection of the donor source of seed.

### Donor and host genetics

The characteristics of the host transgenic mouse strain can be an influential factor in the development of downstream pathology, with different lines of APP transgenic mice responding to seeding by producing Aβ deposits with distinct morphologies and localizations. Autologous seeding may or may not maintain the expected pathology of the models used; for example, APP23 mice injected with APP23 extract from older mice develop primarily diffuse plaques instead of the expected dense core deposits of this model [6, 15] (Tables 1 and S1). APPPS1 seeds injected into APPPS1 mice develop many compact, punctate plaques that are typical of this model [6, 14]. Injecting APPPS1 mice with APP23 seeds results in a mixture of pathology; diffuse, filamentous Aβ as well as compact plaques [6, 14]. Conversely, APPPS1 mice injected with APP23 homogenate develop plaques that are more diffuse than the highly punctate deposits seen in APPPS1 mice injected with APPPS1 homogenate [6, 14]. Localization of seeded deposits is also influenced by the transgenic mouse strain of the donor seed, with APP23- and APPPS1-derived seeds resulting in distinct patterns of plaque localization in the dentate gyrus [14]. Collectively, these findings are consistent with the proposed idea that different pathological morphologies of Aβ deposition are manifestations of a strain-like behavior of the misfolded Aβ that produces these pathologies [35].

To date, it has not been possible to seed pathology in non-transgenic host mice by injecting murine-derived seeds of human Aβ [4, 6, 20, 22]. In addition, the injection of non-transgenic brain homogenate has not demonstrated efficacy in seeding either transgenic or non-transgenic hosts [6–8, 11, 12, 17, 18, 22]. Combining non-transgenic brain homogenate with synthetic Aβ results in limited seeding of Aβ deposition in APP23 mice, but not in non-transgenic mice [6, 11]. These findings indicate that endogenous mouse Aβ is difficult to seed. Overproduction of mouse Aβ by mutant murine APP transgenes produces Aβ deposits that are similar to pathology produced by human Aβ [36], suggesting that mouse Aβ is not inherently resistant to aggregation. The inability to seed non-transgenic mice may be a consequence of the way in which endogenous WT APP is processed by BACE1 to favor the production of Aβ11–40 and Aβ11–42 over Aβ1–40 and Aβ1–42 [37, 38]. Hence, in non-transgenic mice the level of Aβ1–42 may be too low to sustain amyloid deposition even after seeding.

APP processing can lead to a variety of Aβ peptide lengths, which are thought to influence the development of amyloid plaque formation; the main form produced is Aβ40, while Aβ42 (produced at lower levels) is considered pathogenic (reviewed in [39]). Biochemical studies have shown that Aβ42 can form fibrils and aggregates much more rapidly than Aβ40 [40]. To understand the role of these peptides in amyloid pathology, mouse strains with differing levels of each peptide have been developed. These transgenic mouse strains reveal that the levels and ratios of Aβ40/Aβ42, both in the donor and the host, affect the resultant Aβ peptides composition [14]. Through the seeding of Aβ pathology, augmentation of both peptide lengths can be observed; for example, APP23:*Gfap*-luc mice seeded with APP23 homogenate exhibit increases in both Aβ40 and Aβ42 levels [6, 9]. Injection of seeds from APPPS1 mice, with low Aβ40:Aβ42 ratios can lower the Aβ40:Aβ42 ratio in APP23 mice, suggesting that seeded deposits may selectively incorporate specific Aβ peptides [14]. However, injection of seeds from APP23 mice into APPPS1 mice does not significantly alter the Aβ40:Aβ42 ratio, most likely because the host Aβ42 aggregates so rapidly [14]. The most consistent finding is that in APP models that produce both Aβ40 and Aβ42, the deposition of both peptides appears to be induced by seeding.

### Concentration

Another variable that has a clear influence on the development of downstream Aβ deposition is the concentration of Aβ in the seed preparation. Reports of concentrations of Aβ in seeding inoculum are variable; reported as 4.4–9.2 ng/μl [7], 1-10 ng/μl [6], or 10-20 ng/μl [8, 12, 14, 17]. The severity of deposition in seeded APP-transgenic mice generally corresponds to the severity of pathology in the brains used for seeding preparation. In one study of seeding activity across different ages, total seeding activity peaked at the initial stage of deposition (in the donor mice), corresponding to a momentary spike in the Aβ42:40 ratio [21]. Still, seeding homogenates prepared from young APP transgenic donors show lower efficacy due to lower levels of Aβ in the seed preparations [6, 21]. Lowering Aβ concentration in homogenates by immunodepletion predictably attenuates seeding efficiency of the homogenate even when intracerebrally injected [6, 17]. The importance of concentration on seeding efficacy is also evident in peripheral application via intraperitoneal injection, which similarly does not exhibit a 1:1 change in pathology to concentration correlation [17]. Importantly, in models in which investigators have injected seeds peripherally, the levels of transgene expression in APP host mice influences the efficacy of pathology induction [17]. Not surprisingly, the higher the level of expression in the host, the better the efficacy of seeding after peripheral injection.

### Seed preparation and inactivation by treatment

For murine-derived seeds, there are a variety of preparation methods that influence seeding efficacy, such as homogenate preparation via homogenization of whole- or forebrain tissue in PBS (10% w/v) followed by sonication and centrifugation to obtain a supernatant fraction [6, 7, 22]. Extracts may be further diluted in buffer containing bovine serum albumin before injection [9]. Treatments of homogenates that degrade or disrupt the amyloid aggregates diminish seeding efficacy; for example, heating and formic acid treatment attenuate the seeding capacity and terminate seeding ability, respectively [6]. Two methods meant to degrade Aβ aggregates from brain lysates to smaller Aβ assemblies, proteinase K treatment and extended sonication, resulted in slight attenuation and increased amyloid seeding, respectively, with distinct plaque morphology [11]. Proteinase K treated lysates seeded large, congophilic aggregates while longer sonication times resulted in smaller and more punctate amyloid deposits. The treatment of APP23 mouse brain homogenate with proteinase K and subsequent heat inactivation yields seeded mice with lower levels of deposition than that of mice seeded with untreated homogenate; the resulting deposits were primarily diffuse, with a subset of deposits identified as congophilic structures characteristically surrounded by neuritic pathology and gliosis [11]. The murine brain tissue can also be fractionated, with the injection of supernatant and pellet portions of fractionated APP23 homogenate producing 30 and 95% of the level of Aβ deposition, respectively, that was observed by crude homogenates [11]. The level of deposition caused by injection of the supernatant was unexpected given the low levels of Aβ that were present in that fraction [11]. Interestingly, the soluble Aβ seeds were much more sensitive to proteinase K treatment [11]. Increasing the degree of sonication of the Aβ-containing homogenate increases the efficacy of seeding Aβ deposition, while also altering the plaque morphology to smaller punctate deposits [11]. Another aspect of seed preparation is whether the tissue is fresh frozen or formaldehyde-fixed. While formaldehyde-fixed brain homogenate can induce Aβ plaque deposition, the resultant morphology can be changed, such as in the case of APP23 homogenate resulting in diffuse plaques when prepared from frozen tissue, with more punctate deposits resulting when seeds were prepared from fixed tissue [18]. Surprisingly, tissue homogenates dried onto stainless steel wire (implanted into APP23 mice) has been found to seed plaques [7]. Together, these findings indicate that Aβ seeds are relatively stable, can resist inactivating treatments, and can potentially be transmitted on metal surfaces.

### Location of injection

The route of administration of Aβ seed has an effect on the resulting amyloid deposition. Intracerebral injections of murine Aβ seeds are largely focused on the hippocampus, often also spreading to the overlying cortex [6, 7, 9, 11, 12, 14, 18–22]. However, the precise stereotactic location of injections can be altered, resulting in distinct patterns of seeded pathology progression [7]. Injection of Aβ seeds into the olfactory bulb, parietal cortex, entorhinal cortex, striatum, and hippocampus resulted in accelerated seeded pathology that resembled the host animal [7]. Plaque morphology can also be affected by injection location, with striatal injections resulting in more diffuse plaques than in brains injected at other locations (e.g. hippocampus) [7]. Certain aspects of pathology appear to be maintained despite injection location, such as congophilic vascular deposition in APP23 mice seeded by intracerebral injection of APP23 homogenates [7].

Intraperitoneal injection of murine (APP23) brain-derived seed results in seeded deposits which can be both vascular and parenchymal in nature [8, 17]. Additional pathology associated with this intraperitoneal seeding includes glial activation and tau hyperphosphorylation [8]. However, the speed at which intraperitoneal injection of Aβ seed induces pathology is slower than that of intracerebral injection [8]. Intraperitoneal injection, while peripheral, maintains seeding capability in mice that do not express APP peripherally [17]. While intraperitoneal injection can initially result in detectable Aβ in monocytes, liver, and spleen, peripheral deposits are not induced in the long term [17]. Collectively, these studies suggest that the environment in which seeds are introduced may influence the type of Aβ pathology that ultimately develops.

### Morphology of seeded pathology

There are a variety of amyloid plaque-related morphological features observed in Aβ seeded mice that emulate different aspects of Alzheimer’s pathology. Seeded amyloid deposit morphology is influenced by the donor seed; deposits can be either diffuse, compact, or a combination thereof [6, 9, 11, 12, 14, 17, 18, 21]. The induction of Aβ deposition can be associated with astrocytic activation as observed by increased GFAP immunoreactivity [9, 13]. At variable levels, seeded deposits have been reported to stain positive with Congo red [6, 7, 11, 12, 17, 20]. These congophilic deposits can be associated with the vasculature or appear in the parenchyma with glial activation and neuritic pathology [6, 7, 11, 20]. The range of reported pathology includes thalamic cerebral amyloid angiopathy [9, 20]. In the intraperitoneal injection of Aβ seeds, it was observed that large seeded plaques accrue neighboring smaller plaques to a greater degree than would be expected for the host model [17]. These findings clearly indicate the potential to use seeding to direct the type of Aβ pathology that a given APP model may produce; however, in most cases reported, the induction of cored, Thioflavin/Congo red positive deposits is more variable than induction of diffuse Aβ deposits (Tables 1 and S1).

## Seeds derived from aged human brain

The utilization of Aβ seeds from human donors demonstrates a clear avenue whereby amyloid deposition that emulates human pathology could be enhanced. While the variability associated with the utilization of human donor samples is a logical detriment, the ability of human tissue to induce amyloid pathology in APP transgenic mice is clear.

### Donor factors

Human donor-derived Aβ seeding is performed with brain tissue from decedents with diagnosed AD that has been confirmed histologically post mortem. Similar to what is observed in mouse to mouse seeding, human to mouse seeding results in Aβ accumulation at an accelerated time point [4–6, 18, 19]. Homogenates from age-matched human samples that are cognitively normal, but positive for Aβ (likely related to accumulation during aging), also demonstrate seeding capability, though to a notably lesser extent than the Aβ-rich seeds derived from AD-diagnosed donors, 10–15% [4, 6]. Brain homogenates from age-matched controls negative for Aβ deposits, in addition to Aβ-negative young controls, are not found to induce Aβ deposition [4, 10, 18]. Seeding of transgenic APP mice that do not develop amyloid plaques (HuAPPwt) has also been demonstrated using human amyloid-positive donor brain tissue [10]. By contrast, cerebral spinal fluid from AD patients does not demonstrate efficient seeding activity [19].

### Seed preparation

Differential processing of the seeding tissue can be used to attain seeded pathology in vivo. Supernatant fractions from fresh or frozen human AD-diagnosed donor brain homogenate, similar to mouse seed preparation, are commonly used to induce Aβ deposition in transgenic APP-expressing mice [4–6, 10]. In addition to samples that are attained via sonication and centrifugation, tissue can be further processed to yield the formic acid-soluble fraction, which seeds diffuse deposits [19]. Formaldehyde-fixed human AD-diagnosed donor brain-derived seeds result in hippocampal Aβ deposits upon intrahippocampal injection [18]. The seeding of Aβ deposition by hippocampal injection of CSF (whether from AD-diagnosed patients or age-matched human controls), even when concentrated, does not result in Aβ deposition in APP23 mice [19].

### Acceleration of pathology

As observed in the murine-induced seeding of Aβ pathology, seeding with human AD brain homogenates can have a profound influence on level of downstream amyloid accumulation. For example, unilateral intrahippocampal injections of AD brain homogenates into 3 month old Tg2576 mice produced pathology preferential to the injected hemisphere by 8 months of age [4, 5]. In initial reports of this model, pathology was primarily found along the hippocampal fissure and around hippocampal blood vessels [4]. Subsequent studies in which Tg2576 mice that were seeded by human AD brain were aged for 12 months reported exacerbation of cortical Aβ pathology [5]. A striking example of induced pathology by human AD brain homogenates used heterozygous WT human APP mice, which do not develop amyloid deposits [41]. Injection of brain homogenate from a human AD donor into 165 day old WT-APP mice induced Aβ deposits by 285 days post-inoculation [10]. Seeded mice also demonstrated increases in GFAP-positive staining (particularly in the cortex) and some mice developed Thioflavin-S (ThioS) positive aggregates [10]. This study is a clear example in which seeding can be used to generate novel models of AD amyloidosis.

### Morphology of seeded pathology

The morphology of Aβ deposits induced by human donor brain tissue can vary, with a range of important features found. Most aged AD tissue contains numerous diffuse and ThioS positive compact deposits [42]. However, mice seeded with human AD brain homogenates often develop diffuse Aβ pathology, with relatively few ThioS or congophilic compact deposits formed [4, 5, 10, 19]. Human seeding into mouse can produce deposits that favor one Aβ length over another, with some more immunoreactive for Aβ42 than for Aβ40, despite the fact that the host endogenous deposits normally contain both Aβ peptides [4]. A subset of the Aβ pathology includes congophilic deposits, though these may favor Aβ40 aggregation [4]. Overall, similar to studies in which homogenates prepared from mice are used in seeding, the Aβ pathology induced by human seeds is often described as diffuse (Table 1 and S1).

## Seeding with synthetic Aβ peptides

Similar to seeding with brain extracts, injection of aggregates of synthetic Aβ can produce an acceleration in Aβ pathology in APP transgenic recipients [13, 16]. Synthetic Aβ was initially ineffective in seeding amyloid deposition in APP23 mice, even when concentrations were raised 100- to 1000-fold relative to tissue homogenates [6]. Since these initial findings, further studies have demonstrated that bilateral pathology can develop from unilateral injection of high levels of synthetic Aβ seeds, suggesting that pathologically misfolded Aβ can propagate from one hemisphere to the other [13]. Other studies have found that small assemblies of synthetic Aβ, such as protofibrils, and other structures are capable of seeding amyloid deposition in APP transgenic mice [43]. As with other types of Aβ seeds, an array of variables can influence the resultant pathology.

### Seed properties and preparation

In brain homogenate studies, the Aβ40 and Aβ42 content results largely from the genotype and preparation of brain homogenate; in the case of synthetic Aβ seed preparation, the amount of one form versus the other can be controlled. In vitro, synthetic Aβ40 forms longer, straighter fibrils than synthetic Aβ42 [16]. The composition of synthetic Aβ seeds has a large effect on the ensuing plaque pathology, with preparation playing a notable role in this outcome. For example, SDS-treatment of synthetic Aβ42 alters the resultant plaque morphology from numerous, small plaques with more Aβ42 than Aβ40, to larger, less plentiful plaques with an Aβ40:Aβ42 ratio more similar to endogenous APP23 mice (similar in appearance to Aβ40 with and without SDS-treatment) [16]. The deposition of Aβ in these synthetic models is largely along the corpus callosum, with an emphasis on the region neighboring the CA1 in mice injected with synthetic Aβ40 (SDS-naïve) [16]. Seeding with a synthetic dodecamer, termed Aβ42 large fatty acid-derived oligomers, can selectively induce acute cerebral amyloid angiopathy when injected in newborn CRND8 mice [44]. Importantly, seeds prepared from human AD brain can be used to seed synthetic Aβ peptides in vitro [45], and it may be possible to use this method to amplify specific strains of misfolded Aβ for seeding into vulnerable APP mice. The changes in the resulting pathology associated with seeding via synthetic Aβ prepared under slightly different protocols supports the importance of understanding the role of individual components of amyloid seeds in ensuing pathology.

### Morphology of seeded pathology

The seeded pathology from synthetic seeds features pathological morphologies similar to those in other seeding paradigms. Synthetic Aβ injection results in an increase in detected GFAP [13, 16]. ThioS-positive deposits are found in APP23:*Gfap*-luc mice injected with synthetic Aβ40 or Aβ42, with and without SDS treatment [16]. In addition, synthetic Aβ40 injections increase overall levels of Aβ40 and Aβ42 in the host brain compared to uninjected controls [13, 16]. Diffuse or compact plaques can be induced via synthetic Aβ seeding [16]. As the use of synthetic Aβ seeds is refined, it may be possible to more precisely program the type of pathology that forms in the seeded host APP mice.

## How does Aβ seeding compare to prions?

Amyloid seeding shares several features of prion templating, as both proteins develop altered tertiary structure, leading to self-aggregation in the brain (reviewed in [46–49]). Amyloid pathology can be induced via seeding with human homogenates, but requires a host that expresses human APP transgenes and a relatively direct route of seed administration; intraperitoneal administration has demonstrated some induction of aggregate development, while other peripheral methods have not demonstrated efficacy [7, 8]. These external routes that have been tested include oral (administration of brain extract onto tongue over the course of 5 days), intravenous (a dilution injected over 10 days maximum), intranasal (inhalation twice in each nostril), and intraocular (injection into the vitreous cavity), with concentration and dose combinations differing by route of administration [7]. Conversely, oral routes of transmission are an established route of transmission of prion diseases [50]. Experimentally, prion protein cannot induce prion pathology in mice that do not express PrP (knockouts of Prnp), indicating the role of endogenous PrP in the development of pathology [49, 51, 52]. Although prions cannot replicate in Prnp knockout mice, prion infectivity can survive passage through these mice. Similarly, Aβ seeds from transgenic murine brain homogenate can survive passage through APP-null mice [20].

In terms of structure, there is evidence to support the transition in protein structure of Aβ monomer from alpha helices to alpha sheets, and then eventually fibrils in the form of beta sheets [53, 54]. These intermediate assemblies may be recognized as different strains of Aβ with varying seeding abilities. PrP^C^ is also largely believed to be composed of alpha helices, while the prion form, PrP^SC^, has a greater beta sheet composition [49, 55–57]. These conformational changes drive the self-assembly of these the proteins into higher order structures that produce pathological lesions. The relative titer of Aβ seeds in mouse models or humans, does not appear to be as high as typical prion seeding titers. Prion titers are quantified by measuring dilution of seed and assessing incubation time to death. In hamsters infected with prions the infectivity titer routinely measures out at greater than 10^7^, meaning that brain homogenates of an infected animal can be diluted 10 million-fold and still retain sufficient prion seeds to induce disease [58]. In titrating Aβ seeds, the critical measure would be the interval between injection and amyloid deposition since Aβ pathology does not cause obvious clinical signs or death; however, these measurements may be influenced by the location of the injection or the age of the recipient APP model injected (see above). The potency of amyloid seeding is also influenced by the severity of pathology in the donor mouse that is used to produce the Aβ seeds. To compare seeding capability between donor strains, researchers prepared serial dilutions of seed preparations from very old APP23 and APPPS1 mice, and determined the dilutions in which 50% of the animals showed amyloid deposition (SD_50_); the SD_50_ was estimated as 10^3^ and 10^2.57^, respectively [21]. Seeds prepared from old Tg2576 mice could be diluted to 10^6^ and retain seeding capability demonstrating Aβ seeding titers comparable to prions [59]. A direct comparison of the relative seeding capacity of these proteins where the levels of misfolded prion and misfolded Aβ are equivalent in the seeding homogenate would be useful.

## Future directions

The influences of the various variables associated with amyloid seeding demonstrate avenues to manipulate amyloid pathology. One of the consistent benefits of Aβ seeding in murine models is the augmentation of Aβ deposition, which accelerates the time-course of experiments. A future goal of seeding studies could be to identify the molecular mechanisms between the interaction of Aβ and tau, the two hallmark protein aggregates of AD pathology. Seeding studies have the potential to explore the relationship between specific types, or strains, of Aβ pathology and the induction of tau pathology and cognitive decline in vivo.

## Supplementary Information

**Additional file 1: Table S1**. Summary of Aβ seeding studies.

## Data Availability

Not applicable.

## Abbreviations

AD: Alzheimer’s disease
APP: Amyloid precursor protein
Aβ: Amyloid-β
CAA: Cerebral amyloid angiopathy
CNS: Central nervous system
F: Female
GFAP: Glial fibrillary acidic protein
M: Male
PS1: Presenilin1
ThioS: Thioflavin-S
