# Supplementary material for: Remodeling Alzheimer-amyloidosis models by seeding
Source: Mol Neurodegener. 2021 Feb 15;16:8. doi: 10.1186/s13024-021-00429-4 (PMC7885558; doi:10.1186/s13024-021-00429-4)
Supplement: Supplementary file 1 — Additional file 1: Table S1. Summary of Aβ seeding studies. [file 13024_2021_429_MOESM1_ESM.doc]

# Table S1: Summary of Aβ seeding studies

| **Reference** | **Source of seed** | **Host Line** | **Host Onset** | **Host Pathological Features as Described within Publication** | **Age Observed (Age at Time of Injection)** | **Pathological Features of Seed Source as Described within Publication** | **Pathological Features of Induced Aβ Pathology** | **Injection Details** |
| --- | --- | --- | --- | --- | --- | --- | --- | --- |
| (10) | Human (4 donors, 81-91 years, confirmed AD, "superior frontal gyrus or lateral orbital cortex") | Tg2576 mice (male) | 9 months | "Before 12 months of age … discrete, generally dense deposits", both Aβ40 and Aβ42. | 8 months (injected at 3 months) | "profuse senile plaques and neurofibrillary tangles" | Primarily diffuse plaques, largely Aβ42. | Unilateral hippocampal (and overlying neocortical) injection |
|  | Human (1 donor, normal neurologic, 77 years, control, "superior frontal gyrus or lateral orbital cortex") |  |  |  |  | "scattered diffuse, Aβ-immunoreactive" plaques, no NFTs | Some diffuse plaques, though only 10-15% seeding compared to mice injected with human AD seed. |  |
|  | Human (1 donor, normal neurologic, 25 years, control, "superior frontal gyrus or lateral orbital cortex") |  |  |  | N/A | N/A | N/A |  |
|  | Human pre-adsorbed with 6E10 antibody (4 donors, 81-91 years, confirmed AD, "superior frontal gyrus or lateral orbital cortex") |  |  |  |  | N/A | N/A |  |
| (24) | Human (4 donors, AD patient, cortical supernatant extracts) | Tg2576 mice (male) | 8 months no/low Aβ deposition, robust by 15 months | At 15 months, cortex and hippocampus have 2.5-3% area with Aβ deposition | 8 and 15 months (injected at 3 months) | "profuse senile plaques and neurofibrillary tangles" (as in (10)) | For some mice at 8 months, some ipsilateral Aβ-positive gliosis. By 15 months, robust Aβ deposition induced (more robust in ipsilateral entorhinal cortex). | Unilateral hippocampal and neocortical injection |
|  | Human (1 donor, 25 years, control, cortical supernatant extracts) |  |  |  | N/A | N/A (as in (10)) | N/A |  |
| (4) | Human (2 donors, 74 and 85 years old, confirmed AD, "superior frontal gyrus") | APP23 mice (male) | 6 months (neocortex), 9-10 months (hippocampus, males) | Rare deposits 6 months in neocortex. In hippocampus 8-10 months (for males 9-10 months). | 9 months (injected at 5months) | Not described | Significant Aβ deposition induced | Bilateral hippocampal (and overlying cortical) injection |
|  | Human (donor, 95 years, control) |  |  |  | 9 months (injected at 5months) | "sparse Aβ deposits" | Limited to no induced Aβ deposition. |  |
|  | Mouse (APP23, 20-26 months, male and female, neocortex) |  |  |  | 7-9 months (injected at 5months) | Fibrillar, congophilic, Aβ40>Aβ42 | Largely "diffuse and filamentous". Some plaques in parenchyma (and also most plaques around vessels) Congo red positive, exhibiting gliosis (microglia and astrocytes) and dystrophic neurites. |  |
|  | Mouse (APP23, 2 months) |  |  |  | N/A | None | N/A |  |
|  | Mouse (APPPS1, 16 months) |  |  |  | 8 months (injected at 5 months) |  | "course, punctate" |  |
|  | Mouse (APP23 aged, 20 months, immunodepleted) |  |  |  | N/A (6 month old mice injected at 3 months) | None | N/A |  |
|  | Mouse (APP23, 20-26 months, combined with β-1 antibody) |  |  |  | 10 months (injected at 6 months) |  | Decreased "Aβ deposition by > 60%" (vs. ctrl antibody) |  |
|  | Mouse (APP23 aged) OR human AD+ brain, then mouse was treated with β-1 antibody weekly for 3 months starting 1 month post-injection (passive immunization) |  |  |  | 10 months (injected at 6 months, antibody injections weekly from 7 months for 3 months) |  | Inhibition of Aβ deposition >90% |  |
|  | Mouse (APP23 aged) OR human AD+ brain, then mouse was treated with blue carrier protein-Aβ1-17 (active immunization) 1 week, 2 weeks, then every 4 weeks post seed injection |  |  |  | 10 months (injected at 6 months; active immunization injections 1 week, 2 weeks, then monthly) |  | Almost complete inhibition of seeding |  |
|  | Mouse (APP23 aged, treated with formic acid) |  |  |  | N/A | None | N/A |  |
|  | Mouse (APP23 aged, heat treated) |  |  |  | 6 months (injected at 3 months) |  | 45% reduction of Aβ deposition |  |
|  | Synthetic: "soluble or fibrillar… Aβ40, Aβ42, or Aβ40/42" |  |  |  | N/A | None | N/A |  |
|  | Synthetic Aβ with aged wildtype brain extract |  |  |  |  |  | Limited deposition, similar to below |  |
|  | Synthetic Aβ42 at 100-1000X concentration |  |  |  | 9 months (injected at 5 months) | Fibrillar, congophilic | Some deposits in dentate gyrus, amorphous mass (largely injectate) |  |
|  | Aβ from media from APP-transfected cells |  |  |  |  |  | No significant accumulation. Minimal Aβ immunoreactivity (from 7PA2 medium). |  |
|  | Mouse (WT aged) |  |  |  | N/A | None | N/A |  |
|  | Mouse (APP23 aged, Aβ positive) | APPPS1 mice | 2 months (neocortex), 3-4 months (hippocampus) | Some Aβ deposits, largely in the molecular layer of the dentate gyrus | 3 and 5 months (injected at 2 months) |  | Aβ deposits were a "mixture of filamentous and compact types" |  |
|  | Mouse (APPPS1 aged) |  |  |  |  | Aβ42>Aβ40 | "course and punctate deposition" more pronounced than in APP23 mice injected with APPPS1 |  |
|  | Mouse (APP23 aged, Aβ positive) | WT mice | N/A | N/A | N/A | None | N/A |  |
| (13) | Mouse (APP23, 22-28 months, male and female) | APP23 mice (male and female) |  | Aβ deposit morphology differences in different brain regions observed endogenously in APP23 mice similar to that seen in mice with brain seed injections. | 3 months post injection (injected at 2-5 months, proximal to injection site), more robust 6 months after injection. |  | Parenchymal Aβ deposition. Vascular congophilic Aβ deposits. | Unilateral olfactory bulb injection |
|  |  |  |  |  |  |  | Parenchymal Aβ deposition. Vascular congophilic Aβ deposits. | Bilateral parietal cortex injection |
|  |  |  |  |  |  |  | Aβ deposition robust with Congo red positive plaques. 6 months after injection, Aβ deposition can be seen "in the molecular layer of the dentate gyrus". Vascular congophilic Aβ deposits. | Bilateral entorhinal cortex injection |
|  |  |  |  |  |  |  | Amyloid deposition diffuse and more mild than that induced by hippocampal and entorhinal injection. 6 months after injection, deposition in the neocortex above the injection site. Vascular congophilic Aβ deposits. | Bilateral striatal injection |
|  |  |  |  |  |  |  | Parenchymal Aβ deposition. Aβ deposition robust with Congo red positive plaques. Vascular congophilic Aβ deposits. | Bilateral hippocampal injection |
|  |  |  |  |  | N/A |  | N/A (Injected at 2-5 months, analyzed 4-8 months later). | Alternative administration routes: mouth, I.V., eye, nose |
|  | Mouse (APP23, 22-28 months, stainless steel wire coated with seed and allowed to dry), some were also heat-sterilized for 10 minutes at 95°C. |  |  |  | 8-9 months (implanted at 4-5 months) |  | Robust Aβ deposition, largely around wire (which were largely congophilic), though more minor deposition found in hippocampus and vessels of the thalamus and pia (unilateral, on the side of the implant). More distant deposits were not congophilic. | Unilateral implant into "hippocampus and overlying neocortex" |
|  | Mouse (APP23, 22-28 months, stainless steel wire coated with seed and allowed to dry, plasma sterilized) |  |  |  | N/A |  | No deposits induced at 8-9 months (implanted at 4-5 months) | Unilateral implant into "hippocampus and overlying neocortex" |
|  | Mouse (WT, age-matched, dry coat on stainless steel wire) |  |  |  | N/A |  | No deposits induced at 8-9 months (implanted at 4-5 months) | Unilateral implant into "hippocampus and overlying neocortex" |
| (14) | Mouse tissue (APP23, 20-27 months, male and female) | APP23 mice (female) |  | Deposits largely parenchymal. | 8-9 months (injected at 2 months), not seen at 6 months (injected at 2 months) |  | Deposits largely cerebral amyloid angiopathy (CAA), carrying into nearby parenchyma. Vascular amyloid deposits Congo red-positive, and surrounded by "diffuse, Congo red-negative Aβ deposits". Associated with increases in gliosis (astrocytic and microglial) | Intraperitoneal injection (2x 100ul, 1 week between injections) |
| (20) | Mouse (APP23 aged ~31-mo) | Tg(APP23:Gfap-luc mice) (male and female) | 12 months (Milder Aβ deposition seen at same age in un-inoculated mice) | Minor, compact | 12 months (injected at ~2 months) |  | "large numbers of small Aβ plaques as well as more diffuse Aβ deposits". Increased GFAP. | Unilateral hippocampal (and overlying neocortical) injection |
| (25) | Human (1 donor, 90 years, AD diagnosis, cerebral cortex) | human WT APP heterozygotes (HuAPPwt) | N/A | N/A | 450 days, 615 days, 750 days (injected at 165 days) | Not described | Diffuse plaques (450 and 615 days). 3/7 mice ThioS positive (750 days). Increased astrogliosis. | Bilateral hippocampal injection |
|  | Human (1 donor, <1 year, control) |  |  |  | N/A | None | N/A |  |
| (15) | Mouse (APP23, 22-28 months, forebrain, male or female) | APP23 mice (male and female) | 7 months in neocortex, 8-10 months in hippocampus (8-9 for females, 9-10 for males) |  | 9 months (injected at 4 months) |  | Strong hippocampal β-amyloid deposition. | Bilateral hippocampal injection |
|  | Mouse (APP23, 22-28 months, forebrain, male or female) treated with proteinase K |  |  |  |  |  | Strong hippocampal β-amyloid deposition. Diffuse and congophilic deposits. Congo red-positive deposits surrounded by activated glia (astrocytes [GFAP+], microglia [Iba1+]) and dystrophic neurites (which were APP+). Amount of deposition induced only 55% compared to non-proteinase K-treated brains. |  |
|  | Mouse/synthetic (WT tissue, aged 22-28 mo, combined with synthetic Aβ fibrils) |  |  |  | N/A |  | No Aβ deposition induced (levels comparable to levels of PBS-injected controls). Tissue analyzed at 9 months (injected at 4 months). |  |
|  | Mouse (APP23, 22-28 months, forebrain, fractionated supernatant, male or female) |  |  |  | 7-8 months (injected at 3-4 months) |  | Deposits largely diffuse and not congophilic. However, if aged to 9-10 months, increase in frequency of congophilic deposits. Achieved approx. 30% of Aβ induction compared to unfractionated tissue (despite low Aβ load of extract). Congophilic deposits accompanied by glial activation (astrocytic and microglial) and neuritic changes as seen in the non-fractionated seed-injected brains. |  |
|  | Mouse (APP23, 22-28 months, forebrain, fractionated pellet, male or female) |  |  |  |  |  | Plaques similar to those seen in brains injected with non-fractionated brain (both diffuse and congophilic deposits). Congophilic deposits accompanied by glial activation (astrocytic and microglial) and neuritic changes as seen in the non-fractionated seed-injected brains. Developed plaques 95% compared to that induced by unfractionated tissue. |  |
|  | Mouse (APP23, 22-28 months, forebrain, male or female, fractionated supernatant treated with Proteinase K) |  |  |  | 9 months (injected at 3 months) |  | Significantly attenuated seeding of Aβ deposition. |  |
|  | Mouse (APP23, 22-28 months, forebrain, extra-sonicated, male or female) |  |  |  | 8 months (injected at 4 months) |  | Increased Aβ deposition compared to standard sonication controls. Deposits small and punctate (compared to non-sonicated seed-injected brain). Some congophilic deposits. |  |
|  | Mouse (APP23 , 22-28 months, forebrain, male or female) |  |  |  |  |  | Filamentous, dense Aβ deposition. Some congophilic deposits. |  |
| (6) | Mouse (APP23, 22-25 months, forebrain) | R1.40 APP mice (homozygous) | ~15 months (3- and 9-mo no deposition, at 15 months 2/6 mice positive) | Some deposits seen at 15 months. | 9 months (injected at 3 months) |  | Diffuse deposits, not congophilic. Similar level of deposition "in the hippocampus and overlying neocortex" to 15 months injected at 9 months. | Bilateral hippocampal (and overlying neocortical) injection |
|  |  |  |  |  | 15 months (injected at 9 months) |  | Diffuse deposits, not congophilic. Similar level of deposition "in the hippocampus and overlying neocortex" to 9 months injected at 3 months. |  |
|  |  |  |  |  | 15 months (injected at 3 months) |  | Largely Congo-negative, some congophilic deposits (only around site of injection) with "hypertrophic astrocytes" (GFAP+) and "enlarged microglia" (Iba1+). Deposits in parenchyma and associated with vessels. More extensive spread of plaques than in other 2 groups. |  |
| (22) | Mouse (APP23 aged) | APP23:Gfap-luc mice |  |  | 330 (visual by IHC) and 385 (significance determined via western blot) days post injection (2 months) |  | Increased Aβ deposition. increased GFAP. | Unilateral intracerebral injection |
|  | Mouse (CRND8 aged) |  |  |  | 300 days post injection (visual by IHC) (2 months) |  | Increased Aβ deposition. Increased BLI. |  |
|  | Mouse (APP23 or CRND8 aged, purified for Aβ aggregates) |  |  |  | 300 days post injection (western blot) (2 months) | Fibrils. 15-20x more Aβ-rich than non-purified. | Increased Aβ deposition. Increased GFAP. |  |
|  | Synthetic WT Aβ40 (~100x Aβ vs regular brain homogenates) |  |  |  | 330 days post injection (western blot) (2 months) |  | Increased Aβ. Increased GFAP. |  |
|  | Synthetic S26C Aβ40 (~100x Aβ vs regular brain homogenates) |  |  |  | 330 days post injection (western blot) (2 months) |  | Increased Aβ. Increased GFAP. |  |
| (5) | Mouse (APP23, 27-28 mo) | APP23 mice (hemizygous, male and female) | 6 months (45) or 6-8 months (5), though in the hippocampus no pathology seen in 7-9 month group | large deposits, "congophilic amyloid cores with diffuse penumbras [and] diffuse Aβ deposition". Aβ40>Aβ42. | 7-9 months (injected 4-6 months) |  | Plaques surrounded by diffuse Aβ. Greater induction of Aβ compared to APP23 mice injected with APPPS1. | Bilateral hippocampal injection |
|  | Mouse (APPPS1, 16-22 mo) |  |  |  | 7-9 months (injected 4-6 months) |  | "punctate, coarse and compact Aβ-plaques" |  |
|  | Mouse (APP23, 27-28 mo) | APPPS1 mice (hemizygous, male and female) | 2-4 months (4), with some aggregates seen in hippocampus at 4-6 months | "[S]mall, compact… highly congophilic Aβ deposits". Aβ42>Aβ40. | 3-4 months (injected 1.5-2 months) and 6 months (injected at 3 months) |  | "diffuse and filamentous" |  |
|  | Mouse (APPPS1, 16-22 mo) |  |  |  | 3-4 months (injected 1.5-2 months) and 6 months (injected at 3 months) |  | "prominent Aβ-deposition" |  |
| (28) | Synthetic Aβ40 (NaP condition) | APP23:Gfap-luc mice | Some deposits 330 day after injection (6-8 weeks), by 2 years Aβ well deposited | Abundant plaques (seen at 2 years); age-matched controls had few plaques. Aβ40:Aβ42 ~1.5. Older animals may have diffuse Aβ42-positive, ThioS negative plaques. | 330 days after injection (injected at 6-8 weeks) | (long straight fibrils, rarely short fibrils) | Increased proteinase K resistant Aβ vs. controls. ThioS-positive plaques. Plaques Aβ40:Aβ42 ~1.5. Compact plaques. | Unilateral intracerebral inoculation |
|  | Synthetic Aβ42 (NaP condition) |  |  |  |  | (long fibrils, mostly short fibrils) | Increased proteinase K resistant Aβ vs. controls. ThioS-positive plaques. More numerous, smaller plaques vs Aβ40 (NaP)-injected mice. Significant increase in astrogliosis vs Aβ40 (NaP)-injected mice. Plaques largely Aβ42 (40:42 ~0.8). Compact plaques and Aβ42-positive, ThioS negative diffuse plaques. |  |
|  | Synthetic Aβ40 (NaP/SDS condition) |  |  |  |  | (long straight fibrils) | Increased proteinase K resistant Aβ vs. controls. ThioS positive plaques. Plaques contain similar amounts of Aβ40 and Aβ42. Diffuse plaques. |  |
|  | Synthetic Aβ42 (NaP/SDS condition) |  |  |  |  | (long fibrils with some twists) | Increased proteinase K resistant Aβ vs. controls. ThioS positive plaques. Plaques contain similar amounts of Aβ40 and Aβ42. Diffuse plaques. |  |
| (16) | Mouse (APP23/APPPS1 mice, 18-30 months, forebrain) | hemizygous APP23 mice (male and female) | No or few deposits by 8-10 months |  | 7-8 months (injected at 1-2 months), more robust 7-8 months after injection |  | Plaques of neocortex largely parenchymal, variability in degree of vascular plaques. 5-15% plaques Congo red positive. | Intraperitoneal injection (2x 100ul, 1 week between injections) |
|  | Mouse (non-transgenic mice, age-matched, forebrain) |  |  |  | N/A |  | N/A |  |
|  | Mouse (APP23/APPPS1 mice, 18-30 months, forebrain) | homozygous R1.40 (male and female) | 10-14 months (no/few deposits) |  | Somewhat at 9-10 months (injected 1-2 months), more robust when 10-12 months after injection. |  | Plaques of neocortex in younger groups largely vascular, but in older groups more parenchymal. Diffuse plaques. |  |
|  | Mouse (non-transgenic mice, age-matched, forebrain) |  |  |  | N/A |  | No induced Aβ deposition. |  |
|  | Mouse (APP23/APPPS1 mice, 18-30 months, forebrain) | hemizygous APP23 mice with murine APP -/- (male and female) | 9-10 months |  | 9-10 months (injected 1-2 months) |  | Aβ deposition similar in morphology, but lower (not statistically significant) than in treatment-matched murine-APP expressing APP23 mice. |  |
|  | Mouse (non-transgenic mice, age-matched, forebrain) |  |  |  | N/A |  | N/A |  |
| (17) | Human (2 donors, frontal cortex ["CERAD C/Braak stage VI"], maintained in PBS with formaldehyde for 1.5-2 years) | APP23 mice (male and female) |  | Large Aβ plaques with Congo red-positive cores and "diffuse penumbras" | 7-8 months (injected 3-4 months) |  | Strong Aβ deposition. | Bilateral dorsal hippocampal injection |
|  | Human ("non-demented control", ["CERAD 0/Braak stage III-IV"], no Aβ deposition in frontal cortex, maintained in PBS with formaldehyde for 1.5 years) |  |  |  | N/A | None | N/A |  |
|  | Mouse (APPPS1 mice, 20-22 months, fresh frozen, diluted to match fixed tissue, forebrain) |  |  |  | 7-8 months (injected 3 months) | Congophilic plaques that are small and compact. | Small, compact, punctate Aβ deposits. |  |
|  | Mouse (APPPS1 mice, 20-22 months, 4.5% formaldehyde-fixed [and cryoprotected in 30% sucrose for 48hr], forebrain) |  |  |  |  |  | Small, compact Aβ deposits. |  |
|  | Mouse (APP23 mice, 25-27 months, diluted fresh frozen, forebrain) |  |  |  |  | Large Aβ plaques with Congo red-positive cores and "diffuse penumbras". | Diffuse plaques. |  |
|  | Mouse (APP23 mice, 25-27 months, 4.5% formaldehyde-fixed [and cryoprotected in 30% sucrose for 48hr], forebrain) |  |  |  |  |  | Small, compact Aβ deposits. |  |
| (23) | Human (multiple donors, frontal cortex, "CERAD C/Braak V-VI", average age 78 years, fresh frozen and then processed to supernatant from formic acid-soluble fraction) | APP23 mice (male and female) | 8-10 months in hippocampus |  | 12 months (injected at 4 months) |  | Diffuse Aβ depositions, noted even with dilution of fraction. | Bilateral hippocampal injection |
|  | Human (AD patient frontal cortex supernatant fraction mixed with AD patient CSF, 1:1) |  |  |  | 10-11 months (injected 3-4 months) |  | Robust Aβ deposition, largely diffuse. |  |
|  | Human (AD patients, CSF from frozen, fresh, or concentrated 15X) |  |  |  | N/A |  | N/A (6-8 months of incubation [injected 3-4 months], 15X samples analyzed solely after 6 months) |  |
|  | Human (control, CSF, as is or concentrated 15X) |  |  |  |  |  |  |  |
|  | Mouse (APP23 mouse, 2 years, CSF) |  |  |  | 11-12 months, 17-18 months, 24-25 months (injected 3-4 months) |  | No Aβ deposition induction | Bilateral hippocampal injection |
| (11) | Mouse (APP23, 24.5-26 months, male) | APP23 mice |  |  | 3-5 months (injected 3-4 months) |  | Aβ detectible biochemically 1-30 days post injection (levels were increased at 30 days, not tested beyond that) | Unilateral hippocampal injection |
|  |  | APP null mice |  |  | (tested up to 30 days post injection, with Aβ detected but decreased over time) |  | Aβ detectible biochemically 1 day post injection, decreased at 7 days post injection, not detected via ECL immunoassay 30 days after injection. Some observed 180 days after with “ultra-sensitive bead-based single-molecule array Simoa technology”. |  |
|  |  | WT mice |  |  |  |  |  |  |
|  | Mouse (APP23 mouse hippocampi seeded 1 and 30 days prior with APP23 brain tissue [aged 24.5-26 months]) | APP23 mice (male) |  |  | 7 and 11 months (injected at 3 months) |  | Some deposits congophilic (which were also "surrounded by activated microglia and dystrophic" neurites). Deposits both parenchymal and vascular. Degree of seeding decreases with primary incubation time. | hippocampal injection |
|  | Mouse (APP null mouse hippocampi seeded 1 and 30 [and 60 and 180] days prior with APP23 brain tissue [aged 24.5-26 months]) |  |  |  |  |  | Minimal seeding at 7 months from the 30+ day post-injection samples. Increase in deposition from 7 to 11 months. Seeding observed from samples with greater primary seed incubation time [e.g. 60, 180 days] showed more mild seeding upon secondary incubation. Some deposits congophilic (which were also "surrounded by activated microglia and dystrophic" neurites). Deposits were both parenchymal and vascular. |  |
| (21) | Mouse (APP23 mice, 2- and 6-months, male) | APP23 mice (male) | 8-9 months (in males); rare at 6 months, notable by 12 months [frontal cortex]); rare plaques 9-10 months in hippocampus (male). | Notable Aβ deposition in frontal cortex by 12 months. 18 months+ "parenchymal Aβ deposits… in most forebrain regions… and cerebral β-amyloid angiopathy". | N/A |  | N/A | Bilateral hippocampal injection |
|  | Mouse (APP23, 12+ months, male) |  |  |  | Robust at 9-10 months (injected 3-4 months) |  | "diffuse and filamentous" Aβ deposition. Seeding ability increases then plateaus with age. |  |
|  | Mouse (APPPS1, 1.2 months, male) |  |  |  | N/A |  | N/A |  |
|  | Mouse (APPPS1, 3+ months, male) |  |  |  | Robust at 9-10 months (injected 3-4 months) |  | "punctate and compact" Aβ deposition. Seeding ability increases then plateaus with age. |  |
| (12) | Mouse (5xFAD mice, 10 months, whole brain) | 5xFAD mice (male) | 4 months (in the hippocampus) | "[D]ense-cored plaques in ... the cortex, thalamus, brainstem, or subiculum", but not in the dorsal hippocampus (near injection site). | 13 weeks initial plaques observed; also observed at 4 months (injected at 7 weeks) |  | Many Aβ plaques in hippocampus and dentate gyrus (not observed in WT controls or WT-injected controls) | Bilateral hippocampal injection |
|  | Mouse (APP23 mice, aged 21 months, whole brain) | APP23 mice (male) | 10 months (in the hippocampus) |  | 9 months (injected at 6 months) |  | Robust Aβ deposition (not observed in controls) |  |
